# A review of brain research on T2DM-related cognitive dysfunction

**DOI:** 10.1515/med-2025-1253

**Published:** 2025-09-19

**Authors:** Qisheng Liu, Shaobing Dai, Xinyue Li, Yaqian Chen, Ni Wang, Jianping Wu, Yang Zhou, Bing Yan, Yaohua Guo, Yurong Liu

**Affiliations:** School of Biomedical Engineering and Imaging, Xianning Medical College, Hubei University of Science and Technology, Xianning, 437100, Hubei, China; Department of Gastroenterology, Xianning Central Hospital, The First Affiliated Hospital of Hubei University of Science and Technology, Xianning, 437000, Hubei, China; College of Innovation and Entrepreneurship, Xianning Medical College, Hubei University of Science and Technology, Xianning, 437100, Hubei, China; School of Public Health and Nursing, Xianning Medical College, Hubei University of Science and Technology, Xianning, 437100, Hubei, China

**Keywords:** type 2 diabetes mellitus, cognitive dysfunction, research status, brain structure, neural circuits, imaging

## Abstract

**Purpose:**

This article summarizes the brain research progress and main research techniques of type 2 diabetes mellitus (T2DM) combined with cognitive dysfunction in recent years, aiming to provide new ideas for the mechanism research and treatment of cognitive dysfunction in diabetes.

**Methods:**

We performed a systematic literature search using the Google Academic database and the PubMed database and then preparing the manuscript.

**Results:**

Cognitive impairment in patients with T2DM is linked to multiple structural alterations in the brain. These alterations encompass cerebral atrophy, vascular damage, increased white matter hyperintensities, microbleeds, a reduction in gray matter volume in the cerebellar cortex, modifications to the structure of the cerebellar dentate nucleus, and frontal cortex damage. Moreover, it may result in neuronal apoptosis and injury, a decline in the generation and maturation of neurons, disrupted or weakened neuronal autophagy, among other consequences. Investigators are employing sophisticated methods such as diffusion tensor imaging, diffusion kurtosis imaging, resting-state functional magnetic resonance imaging, cerebral blood flow examinations, and voxel-based morphometry to investigate these affected brain areas.

**Discussion:**

The pathogenesis of T2DM-related cognitive dysfunction is not fully understood. This article reviews recent advances in the study of T2DM-related cognitive dysfunction and highlights key research methodologies, offering new insights into the mechanisms and potential treatments for cognitive impairment in diabetes. This review provides a new direction for the study of the mechanism and treatment of cognitive dysfunction in diabetes.

## 1 Introduction

**Figure 1:**
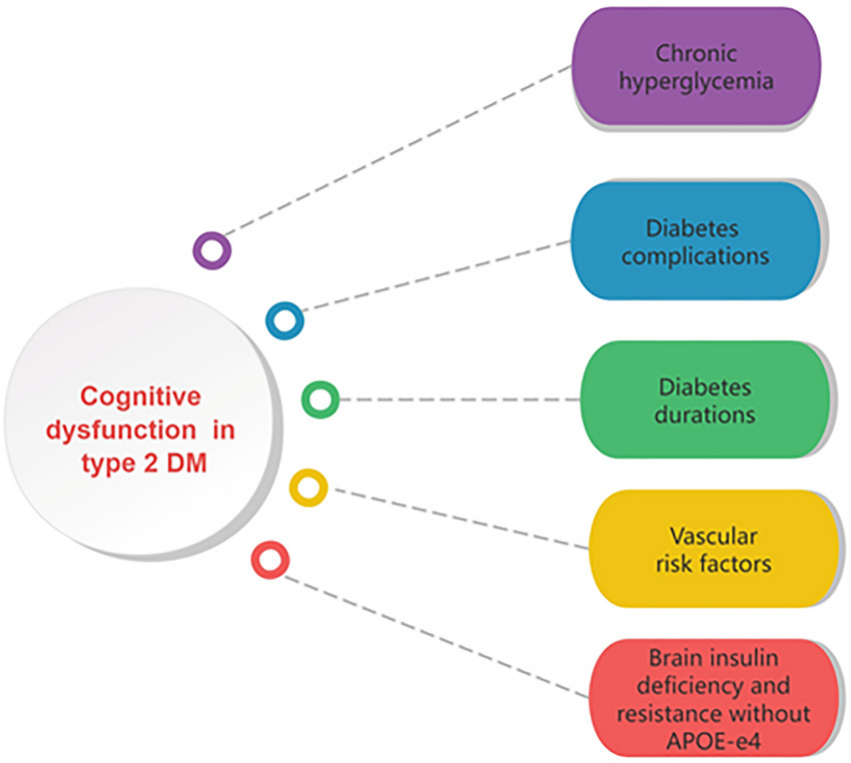
Elements influencing cognitive impairment in T2DM include persistent hyperglycemia, extended duration of the disease, the existence of vascular risk elements such as hypertension and obesity, as well as complications from both microvascular and macrovascular diseases. These factors are linked to a higher likelihood of cognitive dysfunction in individuals with type 2 diabetes.

**Figure 2:**
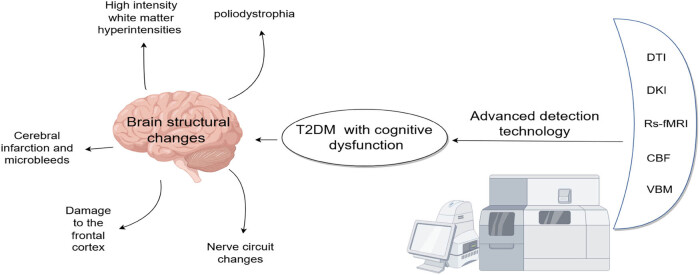
This article explores the various structural and functional changes in the brain associated with type 2 diabetes, outlines the mechanisms of diabetic cognitive impairment through an in-depth study of advanced imaging techniques and recent findings, and highlights potential areas for further research. Figure support was provided by Figdraw.

Diabetes mellitus (DM) is a prevalent chronic metabolic disease characterized by disorders of glucose metabolism. With the rise of an aging population and changes in lifestyle, the number of individuals diagnosed with diabetes has been increasing annually, with a notable trend toward younger onset. Data from a national study carried out from 2018 to 2019 indicate that the general occurrence of diabetes in adults over the age of 18 stood at 12.4%. The frequency was found to be 11.5% among females and 13.3% among males [1]. The International Diabetes Federation calculates that globally, there are approximately 415 million individuals living with diabetes, of which 90% suffer from type 2 diabetes [2]. By 2045, it is projected that the global diabetic population will reach 783 million [3]. Complications of diabetes not only damage large blood vessels and microvessels in the heart, brain, kidney, and nervous system but also cognitive impairment (Figure 1). This review discusses recent advances in research on cognitive impairment associated with diabetes. Figure 2 shows the general structure of the content of this article.

Given the significant impact of diabetic complications on cognitive function, we will next delve into the current research status of diabetes-related cognitive impairment both domestically and internationally.

## 2 Research status domestically and internationally

**Figure 3:**
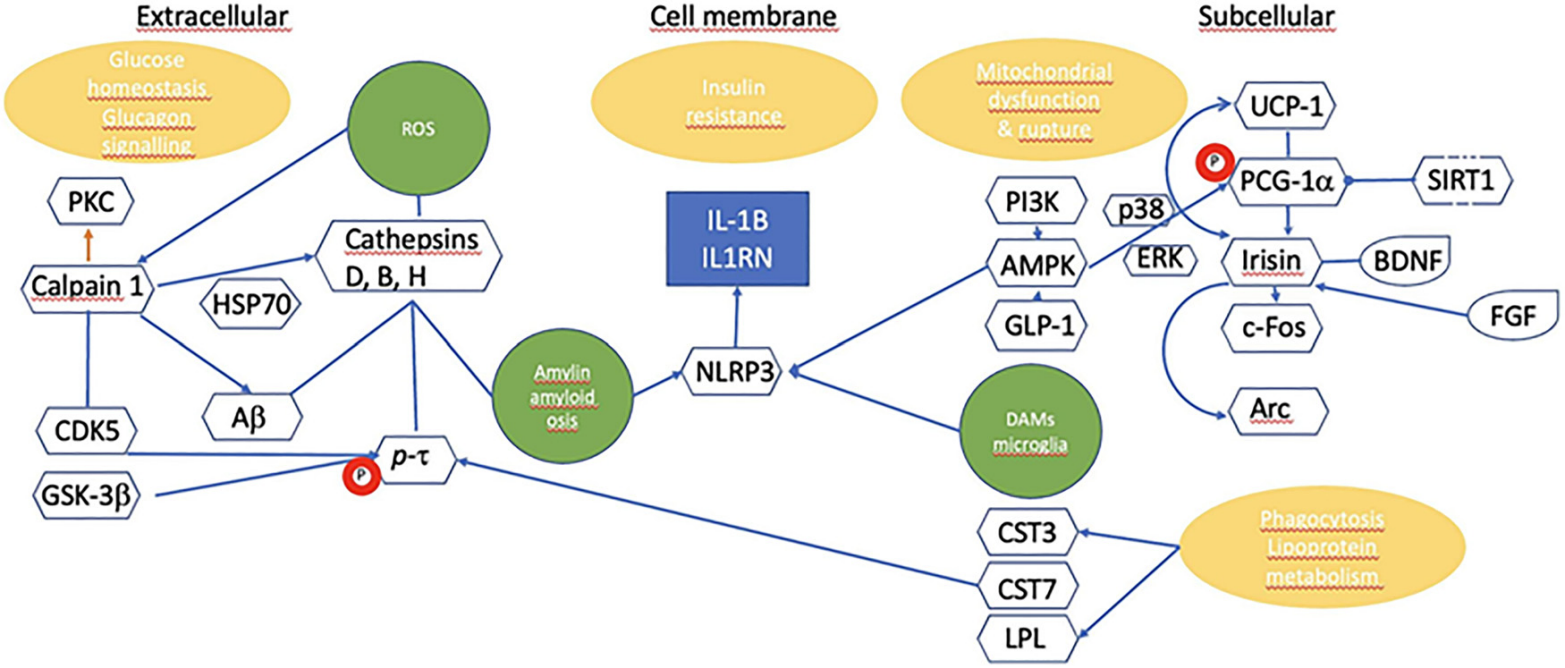
This schematic illustration shows the interaction of different components in brain insulin resistance across the extracellular space, the cell membrane, and the intracellular compartment. Reproduced with permission from ref. [6]. Copyright 2024, Elsevier.

Studies have shown that approximately 60–70% of diabetic patients experience mild to moderate cognitive dysfunction [4]. Cognitive dysfunction in diabetes, particularly in type 2 diabetes mellitus (T2DM), is primarily linked to brain tissue damage, often manifested as memory and learning difficulties. Epidemiological studies indicate that individuals with T2DM are 60% more likely to develop Alzheimer’s disease (AD) compared non-T2DM individuals [5]. Recent research suggests that cognitive impairment in T2DM is associated with insulin resistance and amyloid-beta deposition [6,7] (Figure 3). The brain changes observed in these patients include cerebral small vessel lesions, white matter lesions, altered cerebral perfusion, changes in brain volume, connectivity, and metabolism.

In the past few years, researchers from around the globe have directed their attention to how alterations in neural circuits influence motor control in individuals with diabetes. Ferris et al. have observed that diabetes causes harm to the sensorimotor areas of the central nervous system, subsequently impairing key motor control pathways that involve the cerebral cortex, cerebellum, and basal ganglia. They emphasize that when studying movement disorders in diabetic patients, it is crucial to consider complications in the central nervous system rather than focusing solely on the peripheral nervous system [8]. However, due to the unclear pathogenesis of cognitive impairment in diabetes, there is currently no effective treatment for this condition [9].

In summary, current research has already revealed the close connection between T2DM and cognitive impairment, and emphasized the necessity of further exploring its neurobiological basis. Next, we will delve into the specific structural changes in the brains of T2DM patients to uncover their potential links to cognitive impairment.

## 3 Brain structural changes associated with cognitive impairment in T2DM patients

### 3.1 Brain atrophy and gray matter changes in T2DM

Brain imaging studies of T2DM patients reveal several structural changes, including brain atrophy, cerebrovascular disease, and alterations in brain microstructure, with brain atrophy being the most significantly associated with T2DM [10]. Two MRI studies conducted over time have revealed an accelerated pace of general brain atrophy, especially in those with reduced cognitive abilities, without a notable rise in white matter hyperintensities (WMHs). Type 2 diabetes is linked to reduced gray matter volumes, whereas white matter volumes seem to remain largely unchanged. Furthermore, the volume of ischemic lesions across the brain increases in individuals with T2DM. These indicators are related to cognitive impairments due to diabetes but do not completely account for them [11].

**Figure 4:**
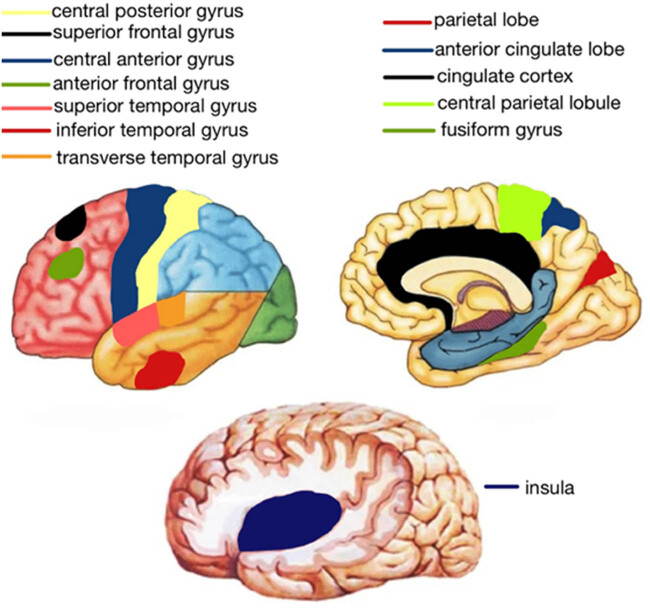
Schematic representation of the brain regions.

Hirabayashi et al. [12] indicate that T2DM is primarily linked to volume reduction in several regions: the temporal lobe cortex (including fusiform gyrus, inferior temporal gyrus, transverse temporal gyrus, superior temporal gyrus, and entorhinal cortex), frontal lobe cortex (central anterior gyrus, superior frontal gyrus, anterior frontal gyrus, and lateral orbitofrontal lobe), parietal lobe cortex (central posterior gyrus, central parietal lobule, and anterior cingulate lobe), occipital lobe cortex (parietal lobe), cingulate cortex (posterior cingulate gyrus and cingulate gyrus), insula, and subcortical gray matter nuclei (putamen, thalamus). These findings are consistent with previous studies, which also reported that gray matter atrophy is distributed across the temporal lobe, frontal lobe, precuneus, cingulate gyrus, insula, caudate, and putamen regions (Figure 4).

### 3.2 WMHs in T2DM

**Figure 5:**
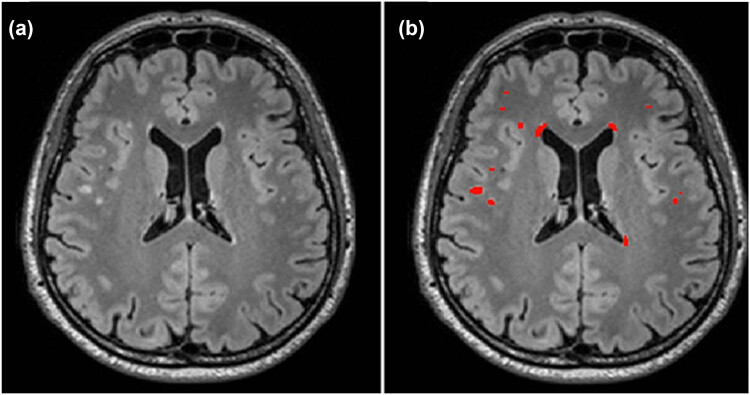
Illustration of manual segmentation for WMHs (total WMH volume: 1,835 mm^3^). (a) Axial T2-weighted FLAIR image from a study subject in his fifth decade. (b) Segmented WMH volume is highlighted in red. The segmentation was performed using ITK-SNAP version 3.6.0. WMH refers to white matter hyperintensity. Reproduced with permission from ref. [13]. Copyright 2021, BMJ Journals.

WMHs are high-intensity areas on MRI scans associated with an increased risk of stroke, cognitive decline, and depression. Identifying the risk factors for WMHs is crucial. Beyond age and hypertension, WMH is significant in both prediabetes and diabetes [13] (Figure 5). T2DM patients often exhibit an abnormal increase in WMHs, which correlates with a higher cardiovascular risk, larger WMH volumes (indicative of white matter dysfunction), slower processing speeds, and attention deficits [14].

### 3.3 Cerebral infarction and microbleeds in T2DM patients

Studies conducted by Manschot et al. [15] and Imamine et al. [16] indicate that stroke in individuals with T2DM is correlated with mild cognitive deficits, specifically affecting processing speed, attention, and executive functions. Several longitudinal investigations have also demonstrated a connection between T2DM and the occurrence of stroke [17,18]. Thacker et al. [19] and associates discovered that high levels of insulin resistance and increased fasting plasma glucose are major risk factors for stroke in people without diabetes. Microbleeds, a type of cerebral small vessel disease, are commonly thought to be linked to cognitive decline, particularly in AD. Nevertheless, research on the association between microbleeds and T2DM is sparse. Studies generally agree that the incidence of microbleeds in T2DM patients does not significantly differ from that in healthy individuals [20].

### 3.4 Frontal cortex damage in T2DM

The frontal lobe is a key component of the cognitive control network [21]. Cognitive control, which is primarily the responsibility of the prefrontal cortex (PFC), is supported by a network of interconnected PFC regions across the brain [22]. Research indicates that cognitive deficits in T2DM are linked to structural harm in the brain, especially in areas crucial for cognitive regulation, like the frontal cortex [21]. Zhang et al. reported that T2DM patients with cognitive impairments had lower density in the medial frontal cortex when compared to healthy individuals, implying a connection to cognitive deterioration [23]. Chen et al. found that under conditions of high working memory (WM) demand, a larger number of frontal lobe regions displayed diminished activation, marking the first study to pinpoint specific brain mechanisms associated with WM dysfunction due to diabetes [24]. Additionally, some studies propose that the right frontal cortex could act as a potential imaging biomarker for the early detection of cognitive impairment in T2DM patients [25].

### 3.5 Neural function and signal transmission abnormalities

#### 3.5.1 Neuronal cell apoptosis and injury

Abnormal neuronal activity and disrupted brain network connectivity form the neural basis of cognitive dysfunction in T2DM patients [26]. Neuronal apoptosis and injury of neurons are associated with several pathological processes.

##### 3.5.1.1 Neuroinflammatory response

Prolonged exposure to high glucose levels leads to abnormal lipid metabolism, triggering an inflammatory response and disrupting homeostasis. Studies have shown that the dysfunction of cerebellar-brain default mode network (DMN) and executive control network (ECN) circuits in T2DM patients may contribute to cognitive and emotional abnormalities. High glucose levels can damage the intrinsic patterns of cerebellar-brain ECN circuits [27]. Additionally, chronic hyperglycemia increases the levels of pro-inflammatory cytokines, causing sustained activation of microglia, which ultimately leads to neuronal damage, apoptosis, and cognitive decline [28].

##### 3.5.1.2 Oxidative stress (OS) response

Injury to the blood–brain barrier (BBB) caused by hyperglycemia induces OS in neurons, leading to the release of reactive oxygen species (ROS) in neuronal mitochondria, which triggers apoptosis [29]. Excessive ROS production leads to OS, abnormal activation of brain immune cells (microglia and astrocytes), and the release of many inflammatory factors, which damage hippocampal neurons, and contribute to cognitive dysfunction [30]. Research suggests that stilbene glycosides may improve cognitive dysfunction in diabetic mice by inhibiting diabetes-induced OS [31]. Gamma-aminobutyric acid in the anterior part of the striatum bed nucleus is projected to the arcuate nucleus to mediate the stress response [32].

##### 3.5.1.3 Endoplasmic reticulum (ER) stress

ER stress can regulate autophagy, induce neuronal apoptosis, and promote cognitive impairment. In T2DM, hippocampal injury combined with increased ER stress triggers the unfolded protein response, leading to increased neuronal apoptosis and cognitive decline [33].

#### 3.5.2 Reduced neurogenesis and decreased neuronal differentiation

The number of new neurons and their ability to differentiate are closely related to their capacity for synaptic integration, which is crucial for proper neural circuit formation. Studies on diabetic mice have found that within 2–3 weeks after neurogenesis, approximately 50% of the new neurons die as they compete for synaptic integration [34].

#### 3.5.3 Autophagy function of neurons is decreased or damaged

Neuronal autophagy is a common pathophysiological mechanism in both T2DM and AD, playing a key role in cognitive dysfunction associated with diabetes [35]. Cognitive decline in T2DM is associated with autophagy dysfunction caused by abnormal autophagosome formation and neuronal degeneration. During the early stages of diabetes-associated cognitive decline (DACD), the body upregulates autophagy to alleviate diabetes-induced neuronal apoptosis and inhibit cognitive decline. However, the use of autophagy inhibitors such as 3-methyladenine can aggravate neuronal apoptosis induced by hyperglycemia, further worsening DACD [36].

#### 3.5.4 Dysfunctional neural signal transmission

##### 3.5.4.1 Abnormal synaptic transmission

Synaptic signaling is crucial for proper neural conduction. In T2DM, hyperphosphorylated Tau protein accumulates in neurons, forming neurofibrillary tangles (NFTs), a hallmark of AD. These NFTs interfere with normal neuronal function, leading to impaired synaptic transmission, and disrupted neural conduction.

##### 3.5.4.2 Neurotransmitter abnormalities

Studies have shown a significant reduction in the synthesis and release of acetylcholine in T2DM mice, which contributes to cognitive decline. Additionally, in the hippocampus of diabetic rat models, the expression of NMDA receptors decreases, hindering long-term potentiation (LTP) and leading to deficits in memory and learning [37].

#### 3.5.5 Changes of neurons in the hippocampus

Hippocampal synaptic plasticity is the physiological foundation of cognitive function. The postsynaptic glutamate-NMDA receptor complex regulates the synaptic plasticity of hippocampal neurons and plays a key role in the development of LTP and long-term depression [38]. Transmission electron microscopy studies on T1DM rats have revealed synaptic abnormalities, including reduced synapse numbers, shorter active regions, wider synaptic gaps, and fewer, smaller, and less-defined synaptic vesicles [39]. Persistent hyperglycemia leads to neurotoxic effects, such as the accumulation of advanced glycation end products, which damage synaptic mitochondria, increase OS, and impair hippocampal synaptic plasticity [40].

In addition to changes in brain structure, advanced imaging techniques can also reveal brain structural alterations, monitor cerebral blood flow (CBF) and metabolic abnormalities, capture neural functional and connectivity abnormalities, evaluate the BBB and neuroinflammation, and conduct longitudinal tracking and mechanism verification, thus providing key tools for revealing the neural mechanisms of cognitive dysfunction in T2DM.

## 4 Technology for detecting T2DM patients

T2DM-related cognitive dysfunction is associated with a range of structural alterations in the brain, including atrophy, WMHs, and frontal cortex damage, among others. To further our understanding of these changes and their implications for cognitive function, advanced imaging technologies play a crucial role. These technologies not only help visualize the structural abnormalities but also provide insights into the functional and metabolic aspects of the brain. In this section, we will outline the key techniques currently employed in detecting cognitive dysfunction in T2DM patients and discuss their application value.

### 4.1 Diffusion tensor imaging (DTI)

DTI leverages the movement of water molecules within tissues to assess the integrity of cerebral white matter and analyze white matter fiber bundles. A key diffusion parameter in DTI is mean diffusivity (MD); higher MD values indicate greater damage to white matter fiber bundles [41]. Studies have shown that T2DM patients exhibit significantly higher MD values in both cerebral hemispheres, indicating white matter damage [42]. A meta-analysis identified abnormalities in ten white matter regions, including the corpus callosum, the fibrillar genera of the corpus callosum, the bilateral anterior radial coronary arteries, the bilateral supra-radial coronary arteries, the bilateral cingulate bands, and the bilateral supra-occipital fascicles [43]. DTI provides a valuable tool for the early detection of microstructural brain lesions in T2DM.

### 4.2 Diffusion kurtosis imaging (DKI)

DKI is an advanced form of DTI that measures the deviation of water molecule diffusion from a Gaussian distribution. DKI offers a more precise analysis of tissue microstructures of tissues, including both white and grey matter, compared to traditional DTI [44]. In T2DM patients, DKI studies have detected microstructural abnormalities in cerebral white matter even before cognitive decline becomes apparent [45].

### 4.3 Resting-state functional magnetic resonance imaging (rs-fMRI)

rs-fMRI tracks brain function by assessing blood oxygen level-dependent signals during a state of rest in the patient [46]. This method has gained extensive use in investigating the neuropathological substrates contributing to cognitive decline in T2DM.

Investigations using rs-fMRI have zeroed in on the DMN, which comprises critical brain structures such as the medial PFC, posterior cingulate gyrus, precuneus, sub-parietal lobule, lateral temporal cortex, and hippocampus. Studies have observed that T2DM patients experiencing mild cognitive impairment exhibit a reduction in functional connectivity within various DMN areas, including the right superior temporal gyrus, right middle temporal gyrus, left angular gyrus, left supramarginal gyrus, and the hippocampus, when compared to those without the condition [47,48]. These observations imply that disturbances in DMN connectivity are correlated with cognitive deficits in T2DM, shedding light on the neuropathological mechanisms at work.

### 4.4 CBF studies

CBF quantifies tissue blood perfusion and reflects changes in cerebral hemodynamic, which are regulated by the neurovascular unit to maintain normal neuronal function. Altered CBF can cause neuronal damage and apoptosis [49]. Research indicates that T2DM patients experience inadequate perfusion in brain regions [50] such as the occipital lobe, DMN-related domains, and cerebellum, which may contribute to cognitive dysfunction [51].

### 4.5 Voxel-based morphometry (VBM) studies

VBM, introduced in 1995, is a technique for measuring structural brain changes, initially used in schizophrenia research [52]. Over time, VBM has been refined and is now commonly used to analyze gray matter in various brain disorders due to objectivity and high reproducibility.

**Figure 6:**
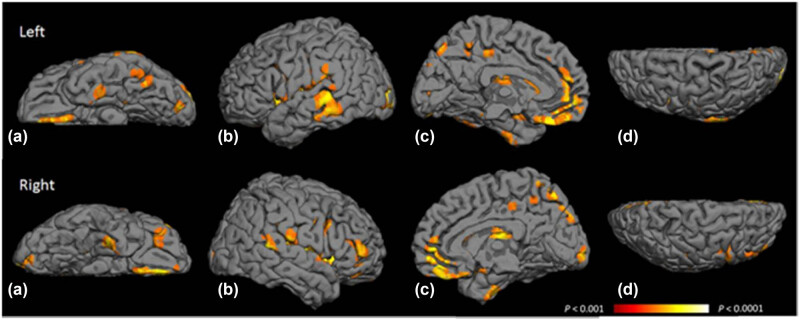
Map depicting the probability of gray matter atrophy due to T2DM. The highlighted voxels represent the brain regions where gray matter atrophy is most probable as a result of T2DM, ranging from a false discovery rate of *P* < 0.001 (depicted in orange) to *P* < 0.0001 (depicted in yellow). A comprehensive list of these areas can be found in Table S1. (a) Inferior area, (b) temporal area, (c) medial area, and (d) superior area. Reproduced with permission from ref. [53]. Copyright 2013, Diabetes Care.

Extensive research has demonstrated that individuals with T2DM exhibit a decrease in gray matter volume, notably in the medial temporal lobe, medial frontal lobe, and anterior cingulate gyrus, alongside a reduction in white matter in the frontotemporal regions [53] (Figure 6 and Table 1). The atrophy observed in the hippocampal area of T2DM patients is directly proportional to the extent of whole-brain atrophy and may be associated with cognitive decline [54]. Studies using VBM have revealed that a decrease in gray matter density within the brain could be an early sign of impending cognitive impairment in those with T2DM [55].

**Table 1:** Associations between T2DM and MRI measures. Reproduced with permission from ref. [53]. Copyright 2013, Diabetes Care. Supplementary information for Table 1 can be found in the Supplementary Material

MRI measures	T2DM (*n* = 350)	No T2DM (*n* = 363)	Association of T2DM with MRI measures^1^	*P* value for regression
Gray matter volume (mL)	579.9 (66.9)	583.4 (63.1)	−13.1 (−18.7 to −7.6)	<0.001
Right hippocampal volume (mL)	2.32 (0.47)	2.77 (0.50)	−0.47 (−0.54 to −0.40)	<0.001
Left hippocampal volume (mL)	2.22 (0.44)	2.61 (0.48)	−0.41 (−0.48 to −0.34)	<0.001
Total hippocampal volume (mL)	4.54 (0.86)	5.38 (0.91)	−0.88 (−1.01 to −0.75)	<0.001
White matter volume (mL)	454.8 (62.1)	456.1 (55.5)	−6.14 (−11.9 to −0.42)	0.05
White matter lesion volume (mL)	6.04 (6.99)	7.10 (8.0)	0.59 (−0.54 to 1.71)	0.32
Infarct yes/no (%)^a,b^	75 (21)	58 (16)	0.62 (0.21 to 1.04)	0.001
Microbleed yes/no (%)^b^	14 (4)	22 (6)	−0.25 (−0.97 to 0.46)	0.41

Data are mean (SD) and *β* (95% CI). ^1^Adjusted for age, sex, and total intracranial volume. ^a^Not adjusted for total intracranial volume. ^b^Not adjusted for stroke history.

## 5 Conclusions

The incidence of DM with cognitive dysfunction is on the rise, often progressing to dementia, which causes significant suffering for patients and their families. This highlights the urgent need for more in-depth research to identify ways to prevent or delay the onset of cognitive dysfunction in DM patients. While brain atrophy has been closely linked to cognitive dysfunction in T2DM, the precise mechanisms remain unclear.

The pathogenesis of T2DM-related cognitive dysfunction is not yet fully understood, and neuroimaging studies exploring changes in brain neural circuits in T2DM cognitive dysfunction are still in their early stages. Some studies have produced conflicting results, indicating the need for further investigation. However, these findings lay the foundation for more extensive and comprehensive future research on T2DM with cognitive dysfunction. Further studies into the specific mechanisms of brain microstructure damage caused by T2DM-related cognitive dysfunction could lead to earlier interventions and improved quality of life of these patients.

## Supplementary Material

Supplementary Table
