# Supplementary material for: A review of brain research on T2DM-related cognitive dysfunction
Source: Open Med (Wars). 2025 Sep 19;20(1):20251253. doi: 10.1515/med-2025-1253 (PMC12452072; doi:10.1515/med-2025-1253)
Supplement: Supplementary Table [file med-2025-1253-sm.pdf]

Supplementary material

Unadjusted and adjusted comparisons of the MRI measures between groups are presented in Table 1. After adjusting for age, sex, and total intracranial volume, T2DM was associated with lower total gray, white, and hippocampal volumes ($P < 0.001$) and the presence of infarct ($P < 0.001$) but not with WMH volume or microbleeds. Restricting the analyses for only the highest quartiles of WMH volume did not alter the findings.

There were 350 people in the T2DM group (mean age 67.8 [SD 6.9] years) and 363 in the non-T2DM comparison group (mean age 72.1 [7.2] years). Group characteristics and comparisons are presented in Table (Table in this document) . Participants with T2DM reported a median disease duration of 7 years (interquartile range 4–12 years). T2DM participants had greater fasting blood glucose levels and HbA_{1c} values, higher BMI and waist–hip ratio, and greater GDS scores and were more likely to report a history of hypertension and hyperlipidemia, treatment with antihypertensive drugs and statins, and lower daily alcohol consumption.

Table S1: Sample characteristics. Reproduced with permission from Reference [1]. Copyright 2013, Diabetes Care

Age (years)	67.8 (6.9)	72.1 (7.2)	<0.001
Female sex	140 (40)	168 (46)	0.09
Formal education (years)	11.3 (3.5)	10.9 (3.7)	0.24
Systolic BP(mmHg)	136.4 (19.1)	141.6 (22)	<0.001
Diastolic BP(mmHg)	76.2 (10.4)	80.4 (11.9)	<0.001
Self-reported history of hypertension or mean systolic BP > 140 or diastolic			
BP > 90 mmHg	252 (72)	163 (45)	<0.001
Use of BP-lowering medications	219 (62.6)	90 (25.7)	<0.001
Ischemic heart disease	82 (23.4)	69 (19.0)	0.15
TIA or stroke	37 (10.6)	24 (6.6)	0.06
Hyperlipidemia	167 (47.7)	32 (8.8)	<0.001
Statin use	218 (62.3)	89 (24.5)	<0.001
Ever smoked	191 (54.6)	179 (49.3)	0.15
Alcohol intake (g/day)	10.8 (16.3)	14.2 (17.5)	0.01
BMI (kg/m^2)	31.1 (8.6)	27.3 (4.3)	<0.0010
Overweight (BMI 25–30)	0 (0)	7 (2)	0.110
Obese (BMI > 30)	129 (37)	183 (50)	0.004
Waist-hip ratio	0.96 (0.1)	0.90 (0.1)	<0.001

(*Continued*)

Table S1: *Continued*

Steps per day	6,013 (3,605)	6,106 (3,185)	0.73
GDS score	2.5 (2.7)	1.9 (2.2)	0.002
Fasting blood glucose (mmol/L)	7.7 (2.3)	5.3 (0.6)	<0.001
HbA_{1C} (%)	7.2 (1.2)	5.6 (0.4)	<0.001
HbA_{1C} (mmol/mol)	55	38	
Age at diabetes diagnosis (years)	57.8 (12.0)	—	—
Median duration of T2DM (years)(IQR)	7 (4–12)	—	—
Insulin use	72 (20.6)	—	—
Cognitive scores (raw,unadjusted measures)*			
Hopkins immediate	23.7 (5.6)	21.8 (6.6)	<0.001
Hopkins recognition	10.2 (1.7)	9.9 (2.0)	0.08
Hopkins delayed	8.1 (2.9)	7.5 (3.1)	0.02
RCFT copy	28.1 (6.5)	31.6 (6.0)	<0.001
RCFT delay	12.7 (6.5)	14.6 (7.1)	<0.001
Digit symbol coding	52.1 (14.4)	49.6 (16.3)	0.03
Symbol search	24.5 (7.6)	22.5 (8.0)	<0.001
COWAT word	35.8 (12.9)	36.2 (13.0)	0.70
COWAT category	18.4 (4.8)	17.0 (5.1)	<0.001

(*Continued*)

Table S1: *Continued*

Digit span	16.1 (4.0)	15.8 (3.9)	0.27
Stroop dot time	16.0 (5.1)	15.6 (5.4)	0.24
Stroop word time	20.2 (6.3)	21.6 (11.2)	0.07
Stroop color time	36.4 (15.4)	37.8 (23.5)	0.36

Data are mean (SD)or *n*(%)unless otherwise indicated .TIA, transient ischemic attack.*Cognitive score comparisons are unadjusted for age,-sex,education,or mood.

References

[1] Moran C, Phan TG, Chen J, Blizzard L, Beare R, Venn A, et al. Brain atrophy in type 2 diabetes: regional distribution and influence on cognition. Diabetes Care. 2013;36(12):4036–42.
